# Expression of MRJP3 and HSP70 mRNA Levels in *Apis mellifera* L. Workers after Dietary Supplementation with Proteins, Prebiotics, and Probiotics

**DOI:** 10.3390/insects13070571

**Published:** 2022-06-24

**Authors:** Maria Carolina Paleari Varjão Oliveira, Eloisa Magalhaes Pereira, Maria Josiane Sereia, Érica Gomes Lima, Breno Gabriel Silva, Vagner Alencar Arnaut Toledo, Maria Claudia Colla Ruvolo-Takasusuki

**Affiliations:** 1 Department of Biotechnology, Genetics and Cell Biology, State University of Maringá, Maringá CEP 87020-900, Brazil; maria.pvo@gmail.com (M.C.P.V.O.); elo.magape@gmail.com (E.M.P.); 2 Department of Engineering and Food Technology, Federal Technological University of Paraná, Campo Mourão CEP 87301-899, Brazil; josiane@utfpr.edu.br; 3 Department of Animal Science, Federal University of Sergipe, Aracaju CEP 49100-000, Brazil; ericalima.zootecnista@gmail.com; 4 Department of Exact Sciences, Luiz de Queiroz College of Agriculture, University of São Paulo, Piracicaba CEP 13418-900, Brazil; brenogsilva@usp.br; 5 Department of Animal Science, State University of Maringá, Maringá CEP 87020-900, Brazil; vatoledo@uem.br

**Keywords:** Africanized honey bee, nutrition, gene expression, heat shock proteins, major royal jelly protein-3

## Abstract

**Simple Summary:**

The development of honey bees depends on royal jelly. Some of the main components of this glandular secretion are proteins known as major royal jelly proteins (MRJPs). In addition to these, bees also produce proteins that help balance the body in situations of stress. During periods of food shortage, beekeepers can supplement colony food to maintain the health and productivity of honey bees. Supplementation can be provided with feed that contains, in addition to other components, probiotics and prebiotics, which are important to balance bees’ intestinal microbiota and, consequently, to keep colonies healthy and without food stress. In order to verify if the addition of probiotics and prebiotics in food supplementation is efficient for the health of bees, after supplementation, we analyzed the levels of synthesis of one MRJP and the stress protein HSP70. The results show that, with the addition of probiotics and prebiotics in the supplementation, there was an increase in the synthesis of the analyzed MRJP3 and low synthesis of the stress protein. Therefore, we suggest that these components be added to honey bee supplementation in periods of food shortage, as it will contribute to colony health in apiaries.

**Abstract:**

Royal jelly is an essential substance for the development of bees from larval to adult stages. Studies have identified a group of key proteins in royal jelly, denominated major royal jelly proteins (MRJPs). The group currently consists of nine proteins (MRJP1–MRJP9), with MRJP1 being the most abundant and MRJP3 being used as a microsatellite marker for the selection of queens with a greater production of royal jelly. The diet of bees is mostly composed of proteins, and supplementing this intake to encourage a higher production of their primary product is important for producers. It is estimated that, by adding probiotic and prebiotic organisms to their diets, the benefits to bees will be even greater, both for their immune systems and primary responses to stress. Circumstances that are adverse compared to those of the natural habitat of bees eventually substantially interfere with bee behavior. Stress situations are modulated by proteins termed heat shock proteins (HSPs). Among these, HSP70 has been shown to exhibit abundance changes whenever bees experience unusual situations of stress. Thus, we sought to supplement *A. mellifera* bee colony diets with proteins and prebiotic and probiotic components, and to evaluate the expression levels of MRJP3 and HSP70 mRNAs using qRT-PCR. The results revealed that differences in the expression of MRJP3 can be attributed to the different types of feed offered. Significant differences were evident when comparing the expression levels of MRJP3 and HSP70, suggesting that protein supplementation with pre/probiotics promotes positive results in royal jelly synthesis carried out by honey bee nurses.

## 1. Introduction

*Apis mellifera* bees are insects with economic and biological significance. Besides their potential as pollinators, they easily adapt to various floral structures, significantly increasing the production of fruits and seeds while producing natural substances with high economic value themselves, such as honey, wax, propolis, and royal jelly [1,2].

Royal jelly (RJ) is essential to the development of *A. mellifera* bees, as it contributes to the differentiation of larvae into queens and, subsequently, remains their only food during the adult phase [3,4,5]. Among RJ components, major royal jelly proteins (MRJPs) first began to be identified in the 1990s, with the primary and most abundant being MRJP1, identified by Hanes and Šimúth (1992) [6,7]. MRJPs account for 82–90% of the total royal jelly proteins [3]. Currently, nine MRJPs (MRJP1–MRJP9) have been described, and their encoding genes (*mrjp1*–*mrjp9*) have been identified in the honey bee genome [8,9].

We demonstrated in a study performed by Baitala et al. [4] that Simple Sequence Repeat (SSR) polymorphisms occurring at the *mrjp3* locus can be used as markers for the selection of RJ-producing queens [4,10]. These polymorphisms were evaluated using analyses of multiple linear regressions with expected progeny difference (EPD) values for RJ production. The variance analyses demonstrated that the *mrjp3* SSR region influenced the genetic value of a queen’s offspring for RJ production. In this study, we also verified that the significant alleles of the repetitive region of *mrjp3* that are responsible for 36.85% of EPD variation are explained by the variation in *C*, *D*, and *E* alleles. We concluded that these three *mrjp3* SSR alleles have a considerable genetic effect on the variation in RJ production.

Generally, bees are exposed to a variety of stressful situations, which include food restriction, diseases, and temperature variation. Such exposures alter bee metabolism, activating biochemical defense mechanisms and triggering reactions at the organizational level [11].

Among the primary cellular protection responses to stress, the expression of heat shock proteins (HSPs) or the heat shock response (HSR) [12,13] stands out. Genes related to HSPs are observed in most species, including bees, demonstrating that their expression is essential for the proper functioning of organisms [14].

The primary responses of organisms tend to be altered according to either the status of the immune system or according to exogenous stimuli. Such interferences can increase or decrease the levels of HSP gene expression [14]. A diet enriched with prebiotics and probiotics could help in obtaining more satisfactory responses involving HSP gene expression while also contributing to the expression of the genes of the main proteins of RJ, such as MRJP3, and, consequently, to the immune system of bees.

In bees, artificial supplementation can positively increase immune responses, in addition to stimulating the production of RJ, as it supplies the nutritional constituents that, at some points during the year, can be crucial for bee development and the maintenance of their colonies [15].

A diet rich in prebiotics and probiotics can significantly increase host health-related benefits. Probiotics, composed of non-digestible beneficial bacteria, can have advantageous effects on their host [16,17]. Feeds that contain probiotic agents are used for the population control of pathogenic microorganisms that may be present in the body of the host, improving the expression of genes related to immunity, that is, mainly those associated with inflammatory responses in various animals, such as poultry, cattle, goats, insects, and humans [15,17,18,19].

However, studies regarding prebiotic and probiotic feeds for *A. mellifera* are scarce. Thus, this study aimed to supplement the diet of *A. mellifera* bee colonies with proteins, and prebiotic and probiotic foods and, subsequently, to evaluate the expression levels of MRJP3 and HSP70 mRNAs using qRT-PCR.

## 2. Materials and Methods

### 2.1. Insects and Experimental Design

*Apis mellifera* worker bees used for this study were collected directly from the brood frames of six commercial Langstroth hives in an experimental apiary at Fazenda Experimental Iguatemi (FEI), in Maringá, Paraná, Brazil (23°24′40″ S; 51°56′23″ W). Bee collection was performed in an entirely randomized manner.

The capped brood frames were removed from the hives and kept in an incubator under controlled conditions for approximately 24 h to ensure the correct age of bees to be used in the experiment. This control made it possible, soon after emersion, to take the workers and isolate them in experimental cages.

In each experimental cage, 125 newly emerged workers were placed according to a protocol pre-established by Williams et al. (2013) [20], totaling 12 cages.

For feed, pollen (2 g), syrup (20 mL), basic protein feed (BPF) (3 g), protein feed with prebiotics and probiotics (FPP) (3 g), and drinking water (30 mL) were used [15,21].

The first treatment consisted of a negative control (pollen, syrup, and water); the second was termed as a positive control (complete set of available food: pollen, syrup, BPF, FPP, and water); the third group of cages consisted of bees that were fed pollen, syrup, water, and FPP; and, in the fourth group of cages, bees were fed pollen, syrup, water, and BPF. All treatments were repeated three times.

All cages were kept in an incubator of the biological oxygen demand (BOD) type, Marconi^®^ thermostatic cabinets with a digital thermostat (supplier Marconi, Piracicaba, Brazil), under controlled conditions of humidity (70% ± 10%) and temperature (32 °C ± 2 °C).

The *A. mellifera* worker bees were kept in the experimental cages for eleven days (from the time of caging until the collection of the last sample), and three collections were completed during this period. Collections were performed on the 4th, 7th, and 11th days after treatments to allow for the development of the hypopharyngeal and mandibular glands so that the expression of *hsp70* and *mrjp3* genes could be evaluated using qRT-PCR and so that mRNAs could be quantified.

A total of 20 worker bees were removed from each collection, and, to facilitate storage, they were subdivided into two samples of 10 workers each; finally, they were stored in Falcon tubes containing Trizol^®^ (Invitrogen, Carlsbad, CA, USA). All samples were stored in a freezer at −20 °C.

### 2.2. Total RNA Extraction and cDNA Synthesis

Total cellular RNA was extracted using Trizol^®^ according to the manufacturer’s guidelines at a ratio of 1 mL to each 100 mg of tissue.

Initially, approximately 0.33 g of tissue from the head and thorax of the bees that were preserved in Trizol was ground. The tissues were crushed until no visible chunks remained, and then 200 µL of chloroform was added. Then, the material was centrifuged for 15 min at 16,128× *g* at 4 °C. The liquid phase was collected and transferred to clean tubes, and 500 µL of isopropanol was added to each tube. After homogenization, the samples were centrifuged for 15 min at 16,128× *g* at 4 °C. The supernatant was discarded, and the precipitate was washed with 1 mL of cold 75% ethanol and centrifuged again at 16,128× *g* for 5 min. After discarding the supernatant and drying for 15 min, the pellet was resuspended in RNAse-free ultrapure water.

The concentration of the extracted RNA was measured with a spectrophotometer at a 260 nm wavelength using Life Technologies Qubit^®^ 2.0 Fluor equipment and the Life Technologies Molecular Probesby Qubit^®^ RNA Assay Kit (500 assays) (Invitrogen, Carlsbad, CA, USA), according to the protocol recommended by the manufacturer.

For cDNA preparation, the SuperScriptTM III First-Strand Synthesis Super Mix (Invitrogen, Carlsbad, CA, USA) kit was used according to the manufacturer’s guidelines. The samples were stored at −20 °C until use. After cDNA synthesis, quantification of the material was performed using Qubit^®^ 2.0 Fluor (Life Technologies, Carlsbad, CA, USA), as previously described.

### 2.3. Real-Time Polymerase Chain Reaction

For real-time PCR (qRT-PCR), the fluorescent compound SYBR GREEN (SYBR^®^ GREEN PCR Master Mix; Applied Biosystems, Carlsbad, CA, USA) and the StepOnePlus™ Real-Time PCR System were used. The primers used in the reactions were designed using www.idtdna.com (accessed on 2 June 2019), and sequences were deposited in the National Center of Biotechnology Information (NCBI) database [Actin (*ACTB*)-AB023025.1, MRJP3-NM_001011601.1, HSP70-NM001160072.1] (Table 1). Actin was used as an endogenous control [22], and the reactions were performed in final volumes of 25 µL.

### 2.4. Statistical Analysis

Data were analyzed using R software v.4.0.2 [23]. Graphics were generated using the ggplot2 package [24]. The hypotheses of normality and the homogeneity of variances of the variables were verified using the Shapiro–Wilk and Bartlett tests, respectively. The F test of the analysis of variance was applied to identify differences between treatments. After observing statistical significance in the F test of the analysis of variance, Tukey’s test was employed. A significance level of *p* < 0.05 was considered in all tests.

## 3. Results

The MRJP3 mRNA presented, on average, higher levels of expression on the 11th day of the experimental period in the negative control, positive control, and FPP treatments.

**Figure 1:**
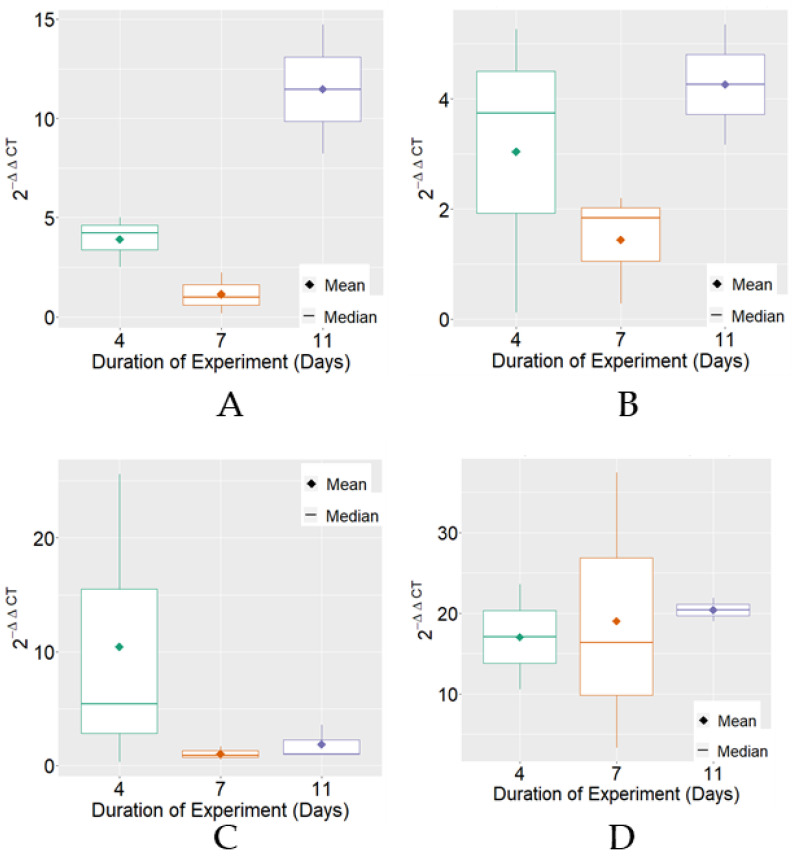
Boxplot of variable 2^−ΔΔCT^ for MRJP3 mRNA expression for each treatment. (**A**) Negative Control; (**B**) Positive Control; (**C**) BPF—basic protein feed; (**D**) FPP—protein feed with prebiotics and probiotics.

Considering the expression of MRJP3 mRNA, variable 2^−ΔΔCT^ exhibited, on average, higher levels on the 11th day of the experimental period in the negative control, positive control, and FPP treatments. Regarding variability, differences were found between treatments for the experimental periods that were individually observed (Figure 1).

**Figure 2:**
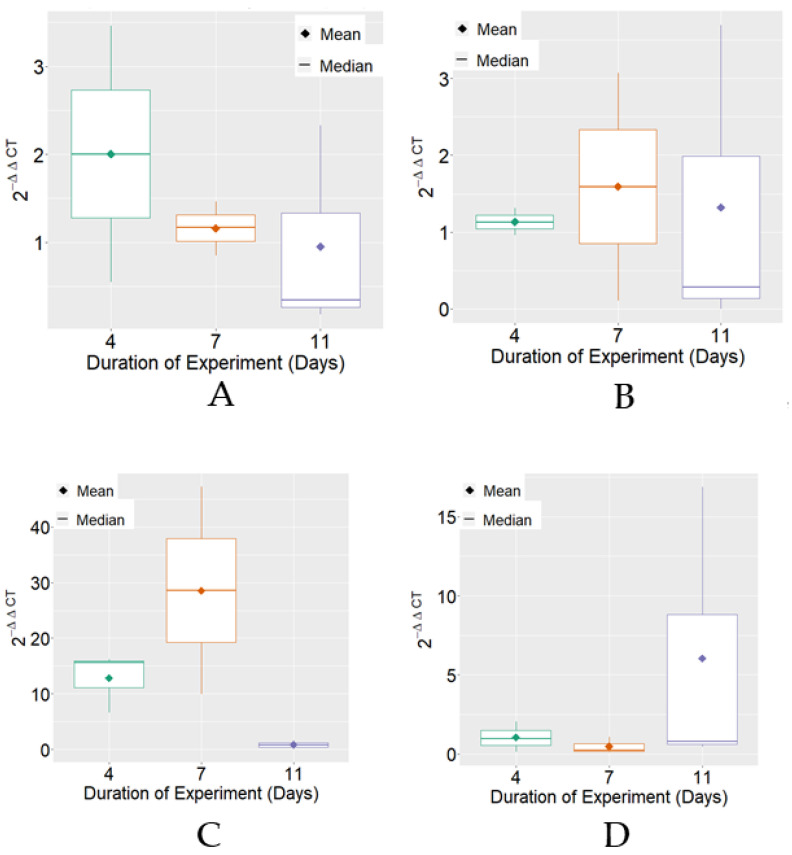
Boxplot of variable 2^−ΔΔCT^ for HSP70 mRNA expression for each treatment. (**A**) Negative Control; (**B**) Positive Control; (**C**) BPF—basic protein feed; (**D**) FPP—protein feed with prebiotics and probiotics.

The expression of HSP70 mRNA, on average, reached the highest levels on the 7th day of the experimental period in the positive control and BPF treatments. Regarding variability, it was verified that there were differences between the treatments for the experimental periods that were individually observed (Figure 2).

**Figure 3:**
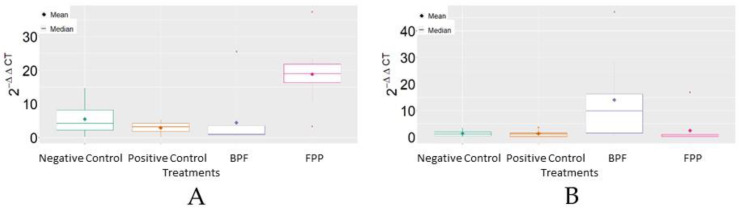
Boxplot of the variable 2^−ΔΔCT^ between treatments. (**A**) MRJP3; (**B**) HSP70. BPF = basic protein feed; FPP = protein feed with prebiotics and probiotics.

Analysis of MRJP3 mRNA expression revealed that FPP, on average, reached the highest levels of 2^−ΔΔCT^ in comparison to the other treatments. For HSP70 mRNA, it was verified that the basic protein feed (BPF) reached, on average, the highest levels of 2^−ΔΔCT^ in comparison to the other treatments (Figure 3).

It was verified by both the Shapiro–Wilk and Bartlett tests that the hypotheses of normality and homogeneity of variance were accepted (*p*-value > 0.05). Table 2 shows the results of the F test of variance analysis. It can be noted that, except for the main effect related to the experimental period, there was statistical significance (*p*-value < 0.05) involving the other main effects, as well as double and triple interactions; i.e., it was necessary to perform the unfolding of the analyzed factors, as can be seen in Table 3.

Regarding variable 2^−ΔΔCT^, it can be verified (Table 3) that, when comparing all treatments, considering each of the experimental periods and proteins used, it is usually concluded that there are significant differences among the treatments (*p*-value < 0.05), with FPP treatment showing the highest averages of the variable 2^−ΔΔCT^ in most of the experimental periods. As for the comparison between the MRJP3 and HSP70 proteins, initially considering the 4th day of the experiment, significant differences were observed after FPP treatment. On the 7th day of the experiment, significant differences were observed between the MRJP3 and HSP70 proteins in the BPF and FPP treatments. Finally, on the 11th day of the experiment, statistically significant differences were observed between the MRJP3 and HSP70 proteins in the negative control and FPP treatment groups.

## 4. Discussion

The highest expression values for MRJP3 mRNA were observed in the negative control analysis of eleven-day-old worker bees. Since this treatment did not involve supplementation with either prebiotics or probiotics, it can be thought that these levels of *mrjp3* gene expression naturally occur in the observed Africanized *A. mellifera*. An increase in the expression of MRJP3 in this same period of development of adult workers was also verified in a proteomic analysis of the development of the hypopharyngeal glands of *A. mellifera* workers performed by Feng et al. (2009) [25]. The authors [25] demonstrated that MRJP1, MRJP2, and MRJP3 expression levels only significantly increased from immediate emergence to day 6 of development and that they decreased after day 12. However, the expressions of these proteins peaked from day 6 to day 12.

Comparative proteomics involving Italian *A. mellifera* workers and RJ producers showed higher expressions of MRJP3 and MRJP4 in RJ producers at the evaluated ages of 1, 3, 6, 12, 15, and 20 days old [26]. The authors accordingly suggested that selection pressure for RJ production had a positive action on these genes. Jianke and collaborators (2010) [26] and Deseyn and Billen [27] also found that the diameters of the acini of the hypopharyngeal glands of workers producing RJ were larger; therefore, they may be directly related to the levels of expression and the production of RJ. In our study, we did not observe the diameter of the hypopharyngeal gland acini, but there was a significant difference in the expression of MRJP3 depending on the supplementation used.

The age-dependent phenotypic plasticity present in *A. mellifera* and its relationship with different expression levels of MRJPs, including MRJP3, were verified by Dobritzsch et al. [28] and Kim et al. [29]. This relationship is partly mediated through natural factors, including gene, age, and tissues analyzed (hypopharyngeal glands and brain). These authors verified high levels of MRJP3 expression in the hypopharyngeal glands of nurse honey bees, as observed in the present study.

The high levels of MRJP3 mRNA expression following FPP treatment (prebiotics and probiotics) allowed us to verify the positive interference of these agents in the development of the treated worker bees. High levels of MRJP3 mRNA expression were also observed in the positive controls. It can be considered that the high levels in the positive controls may be associated with the better food supplementation. In this treatment (positive control), the supplement contained prebiotics, probiotics, and syrup.

In this study, there was a significant increase in *mrjp3* gene expression between FPP treatment and the other treatments (*p*-value < 0.05). The comparisons of the BPF group, negative control, and positive control did not reveal significant differences (*p*-value > 0.05).

With supplementation, *hsp70* gene expression was also analyzed. HSP70 represents a molecular chaperone that is important for proteostasis, acting in the folding, disaggregation, and degradation of proteins [30]. In this study, it was found that there was an increase in HSP70 mRNA levels on the 7th day of the food supplementation of *A. mellifera* nurse workers with protein feed (BPF). This stage corresponds to a period when nurse workers increase their synthesis of RJ [6]. According to the HSP70 model proposed by Fernández-Fernández and Valpuesta [30], these proteins function as a “multi-socket”. Therefore, HSP70 provides a physical platform for the binding of client proteins, other chaperones, and cochaperones. The fate of the client protein is dictated by the set of HSP70 interactions that occur in each given cellular context. Thus, considering the model proposed by Fernández-Fernández and Valpuesta [30], protein feed supplementation may have promoted increased HSP70 mRNA synthesis resulting in HSP70s then acting on client proteins arising from the supplementation and finally contributing to RJ synthesis. This is the first study showing this change in HSP70 mRNA expression in *A. mellifera* in association with MRJP3 expression.

From the results obtained here, it was possible to observe that, following supplementation with prebiotic and probiotic agents, HSP70 mRNA showed a variable expression pattern. As the age of the bee advances, the levels of stress and stressors tend to change. However, the ingestion of prebiotic and probiotic agents improves the immune system, thus supporting the eventual decreases in stress levels and thereby increasing host welfare and causing beneficial biochemical reactions through the expression levels of HSPs [31]. The increased expressions of HSPs suppress the synthesis of nascent peptides that may undergo folding under stressful conditions, preserving the intermediate folded states of incipient proteins until they are depleted or degraded [32].

Black et al. [14] conducted studies where forage bees of *A. mellifera* in their last weeks of life were subjected to electric shocks. Subsequently, differences in the expressions of HSP proteins were identified. When stimuli interfered in a harmful manner, there was an increase in the expression of HSP70 [14]. However, it was observed in this study that, when offered a more nutritious diet, bees exhibited lower expressions of HSPs, suggesting an improvement in the physiological responses of organisms.

At sufficient levels, stress proteins, especially HSP70, allow cells to survive under stressful conditions because they prevent the accumulation of misfolded proteins, protect denatured proteins from proteosomes, and inhibit the caspase-induced apoptosis pathway (proteases that act in cell death or apoptosis) [33]. When stress conditions overcome the cellular protection offered by stress proteins, the inhibition of apoptosis discontinues, and cell death occurs as a means to delete damaged cells and prevent inflammation [11].

Thus, when the results obtained in this study were analyzed, it was observed that supplementation with prebiotics and probiotics can cause a decrease in the expression of HSP70, which might contribute to the modification of proteins involved in the intermediate metabolism of bees, making them less vulnerable to external agents that might otherwise harm the development or functions of the colony.

## 5. Conclusions

Nurse workers of *A. mellifera* supplemented with proteins and pre/probiotics showed an increase in the expression of MRJP3 mRNA and variation in the expression of the stress protein HSP70. These results are initial, but due to the role of MRJP3 in the synthesis of royal jelly and the role of HSP70 in the intermediary metabolism of honey bees, protein supplementation containing prebiotics and probiotics positively contributes to the synthesis of royal jelly.

## Figures and Tables

**Table 1:** Primers used for qRT-PCR and the respective amplicon sizes, annealing temperatures, and sequences.

Gene	Amplicon Size (bp)	Annealing Temperature (°C)	Primer Sequence 5′-3′
Actin Forward	134	60	CCATGTATCCTGGAATCGCAG
Actin Reverse			AGAAGCAAGAATTGACCCACC
*mrjp3* Forward	143	60	TTGACAGTC GCTGGA GAA AG
*mrjp3* Reverse			GTGGATTGCTGAATTGTTCCG
*hsp70* Forward	74	60	CAAGAGAGGAACACGACCATACC
*hsp70* Reverse			AGACGCCAGGTTGATTATCG

**Table 2:** Analysis of variance for the variable 2^−∆∆CT^.

Variation Sources	DF	Mean Square
		^−ΔΔCT^
PE	2	5.10
T	3	320.50 ***
P	1	172.50 ***
PE × T	6	121.40 ***
PE × P	2	142.70 ***
T × P	3	510.50 ***
PE × T × P	6	120.18 ***
Residual	48	42.60
CV (%)	-	102.37
Overall Mean	-	6.37

*** Considered significant if *p*-value ≤ 0.05 by the F test; PE: periods of the experiment; T: treatments; P: MRJP3 and HSP70 proteins; DF: Degrees of Freedom and CV: Coefficient of Variation (%).

**Table 3:** Variable 2^−ΔΔCT^ with the unfolding between the MRJP3 and HSP70 proteins, and between the treatments.

Periods of the Experiment	Treatments	2^−^^∆∆CT^	
		MRJP3	HSP70
4	Negative Control	3.91 aC	2.00 aB
4	Positive Control	3.04 aC	1.13 aB
4	BPF	10.43 aB	12.84 aA
4	FPP	17.06 aA	1.04 bB
7	Negative Control	1.13 aB	1.16 aB
7	Positive Control	1.44 aB	1.59 aB
7	BPF	1.02 bB	28.56 aA
7	FPP	19.03 aA	0.48 bB
11	Negative Control	11.47 aB	0.95 bB
11	Positive Control	4.26 BC	1.32 aB
11	BPF	1.84 BC	0.81 aB
11	FPP	20.41 aA	6.03 bA

Average followed by distinct lowercase letters in the columns and uppercase letters in the rows (*p*-value ≤ 0.05) differ between them according Tuckey’s test.

## Data Availability

The data presented in this study are openly available in the supplementary material.

## Supplementary Materials

The following supporting information can be downloaded at: https://www.mdpi.com/article/10.3390/insects13070571/s1, Table S1: MRJP3 calculation and HSP70 calculation.
